# Framework for developing research and policy to address climate-related occupational hazards

**DOI:** 10.3389/fpubh.2025.1692505

**Published:** 2025-12-05

**Authors:** Paul A. Schulte

**Affiliations:** Independent Occupational Safety and Health Consultant, Cincinnati, OH, United States

**Keywords:** climate change, ambient temperature, workers, environment, public health

## Abstract

Throughout the world, workers are suffering and dying from exposure to climate-related hazards. The threat to workers from these hazards is growing, making worker protection actions an urgent concern. This Policy Brief provides a protective framework. It consists of five linked elements; climate-related hazards are causally linked to adverse effects, which requires needed action and which are modified by various factors. To carry out the needed action, this brief proposes a new emphasis built on the realization that the responsibility for preventing climate-related hazards to workers falls on both employers and authorities/politicians since climate change is not only a workplace issue but also a public issue that requires political action to mitigate exposure.

## Introduction

**Figure 1:**
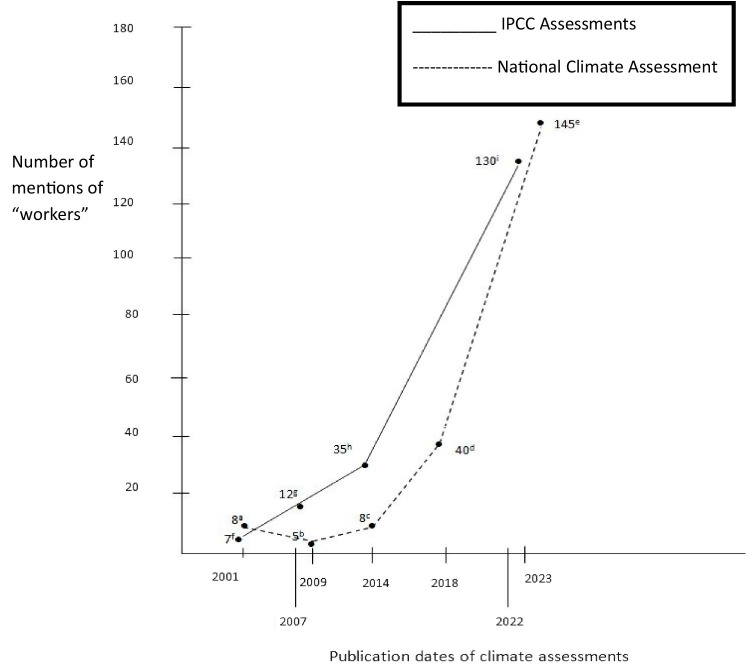
Mentions of “workers” in IPCC reports and US national climate assessments. a. Climate change impacts on the United States: the potential consequences of climate variability and change (72); b. global climate change impacts in the United States (73); c. climate change impacts in the United States (74); d. fourth national climate assessment (75); e. Fifth national climate assessment (76); f. climate change 2001: impacts, adaptation, and vulnerability (77); g. climate change 2007: impacts, adaptation, and vulnerability (2007) (78); h. climate change 2014: impacts, adaptation, and vulnerability (79); i. climate change 2022: impacts, adaptation, and vulnerability (80).

There is increasing awareness that workers are at risk of adverse effects from climate change (1–8). However, this insight was not always the case. Figure 1 shows how many times the term “workers” is mentioned in reports from the Intergovernmental Panel on Climate Change and in US National Climate Assessment reports starting in 2001. In the first few years, workers were hardly mentioned, but use of the term has increased significantly since. In 2009, Schulte and Chun noted the absence of workers in the climate literature and promoted more attention to them (9). Indeed, workers are at increased risk of adverse effects (e.g., physical and mental disorders, traumatic injuries, wage or job loss) from climate change because they are generally one of the first groups in society significantly exposed to climate-related hazards, and their exposure is typically greater than that of the general population (4, 8, 10). Moreover, employers are not generally prepared to protect workers from climate-related hazards. Employers are used to controlling known and well-understood chemical, biological, physical, safety, and organizational hazards in the workplace but not climate or weather ones, and workers have typically not been a part of jurisdictions’ or businesses’ climate-response plans (6, 10). However, with increasing awareness of the risks to workers from climate change, this situation has been changing (8, 11).

Since workers are clearly at increased risk of adverse effects from exposure to climate-related hazards, there is an urgent need to assess and control these hazards and exposures to address this risk. However, countering this need and resultant action, there are significant efforts to attempt to justify inaction or inadequate response (12).

**Figure 2:**
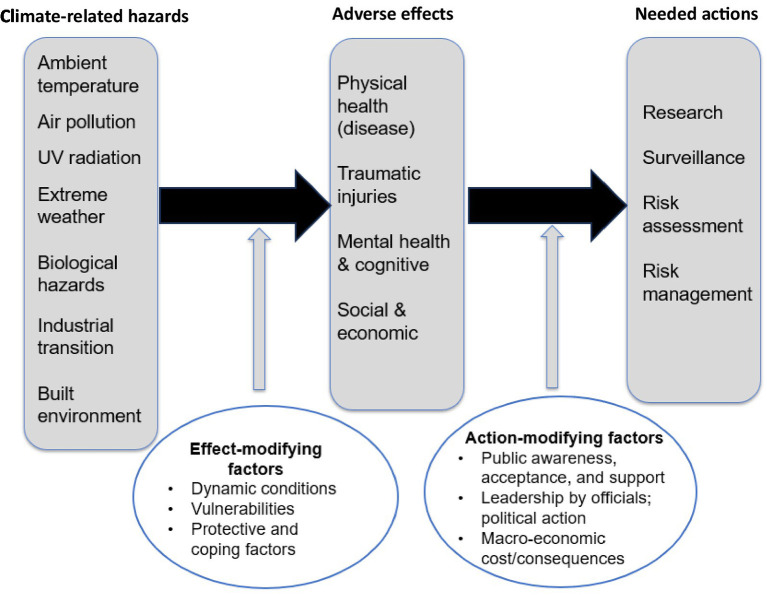
Framework to address the impact of climate change on workers. Adapted from Schulte et al. (10).

To begin to address workers’ risks from climate-related hazards, building on previous work (6, 9, 10) and utilizing a general conceptualization approach (13), I developed an overarching framework (Figure 2). This framework shows that exposure to climate-related hazards results in adverse effects, and this relationship is modified by inherent and related factors (Effect-modifying factors). Ultimately, the risks to workers need to be ameliorated by investigative and control action (Needed actions). However, the ability to mitigate climate-related hazards and the effectiveness of doing so are modified by factors that inhibit the implementation of controls (Action-modifying factors).

## Climate-related hazards

A model to identify climate-related hazards for workers was developed in 2009 and reiterated in 2016 and 2023 (6, 9, 10). This model identifies seven categories of hazards (9). The first five categories are direct hazards and include increases in *ambient temperature*, *air pollution*, *UV radiation*, *extreme weather*, and *biological hazards* (disease vectors and expanded habitats). The other two hazards are indirect and include *industrial transition*, which results in adverse worker impacts from job loss when fossil fuels are phased out and newer industries are created, and *hazards of the built environment*, which occur when constructing or maintaining hard structures like highways, wind turbines, levees, solar farms, and buildings (3, 6, 9). Also, there are many subfactors that affect workers, such as shortages of clean water, or loss of food crops from droughts that are subsumed by the seven categories.

## Adverse effects from climate-related hazards

The adverse effects that can result from exposure to climate-related hazards fall into four categories: physical health effects (disease), traumatic injuries, mental health and cognitive effects, and social and economic effects (2, 3, 6). These different effects may overlap.

### Physical health effects (disease)

Most prevalent among the physical health effects stemming from climate-related hazards are those pertaining to exposure to increased UV radiation and extreme ambient temperature, and air pollution, because they have a broad geographic distribution. Exposure to increased ambient temperature leads to heat-related illnesses and deaths (1, 7). Similarly, exposure to air pollution leads to acute and chronic respiratory and cardiovascular effects in workers (14), and exposure to UV radiation leads to eye damage and skin cancer (15). Climate change can also expand the natural habitats of disease vectors, leading to infectious and dermal effects (6, 10).

### Traumatic injuries

Exposure to various climate-related hazards can lead to traumatic injuries in workers. In extreme heat conditions, workers are at risk of workplace incidents resulting in injuries due to fatigue and heat-related impacts on cognitive processes (16, 17). In addition, outdoor workers have been killed or injured from lightning strikes, which are projected to increase in the 21st century (18). Exposure to ground-level air pollution has also been associated with work-related injuries (19), and traumatic injuries have occurred in workers exposed to extreme weather events (10, 20). Even efforts to impact climate change with sustainable activities, such as recycling, have led to injuries among workers (21).

### Mental health and cognitive effects

Climate-related hazards also have a broad range of adverse effects on workers’ mental health and cognitive health (4, 22–24). For instance, extreme heat exposure can lead to irritability, confusion, decreased motor control, and decreased cognitive performance in workers (25). Further, workers engaged in climate-related disaster response have elevated risks of unhealthy coping, depression, post-traumatic stress disorder, and suicidality (26). Similarly, farmers exposed to droughts have increased risk of suicide, and the expanding habitats of vector-borne diseases, such as West Nile Virus, have been shown to result in depression (27). Even workers who study and document climate disruptions suffer from mental health burdens (28).

### Social and economic effects

In addition to the physical and mental health effects resulting from climate-related hazards, there are a host of social and economic effects. Specifically, exposure to heat has led to decreased capacity and productivity in workers (2). Indeed, it is projected that by 2050, the world may lose 10% of its total economic value from this capacity and productivity loss (29). Moreover, job loss and climate-related migration among workers are currently occurring and could increase overall because climate change is a risk multiplier for workers (30). It is estimated that 19% ($38 trillion USD) of the reduction in global income resulting from climate change will occur by 2049 (31). Moreover, it is estimated that by 2050, “US workers could lose 883 million hours of work from heat (this is roughly equivalent to 423,000 workers sitting idle for a year)” (4).

## Effect-modifying factors

The relationship between exposure to climate-related hazards and adverse effects is well documented but varies across a range of conditions (32). Climate conditions and resultant hazards are variable across space and time. Moreover, as climate change progresses, hazard frequency and severity can increase as well as the prevalence and incidence of adverse effects (33–35). Accordingly, worker protection in changing environments is influenced by the dynamic nature of climate-related hazards. Further, the distribution of these hazards and effects is influenced by various vulnerabilities (e.g., age, obesity, mental illness, medications, pregnancy, and genetics) as well as socioeconomic, demographic, and geographic factors, which can lead to disproportionate burdens on the individuals who are least able to resist them and who have the least protective technologies and coping interventions available to them (9, 30).

## Needed actions

The burdens of climate change on workers require action to ensure they have safe workplaces and working lives. Four categories of action have been identified: research, surveillance, risk assessment, and risk management (10).

### Research

There is a need to further investigate the link between work, health and safety, and climate change, particularly in relation to workers who experience vulnerabilities. In addition, the effectiveness of interventions needs to be evaluated to protect workers from exposure to climate-related hazards and the resultant deleterious effects. Implementation science needs to be supported and utilized because it provides a valuable set of approaches to address the multilateral contextual factors that inhibit the mitigation of and adaptation to climate-related hazards (36). Additionally, the ability to anticipate the effects of the climate on workers needs to be improved. Also important is the need to investigate how to combat climate-change misinformation (inadvertent spread of false information), disinformation (intentional spread of false information), and political resistance (discussed later in the brief) because these factors serve as a barrier to need action.

### Surveillance

Surveillance is critical to help ascertain the burdens climate change creates for workers, including the most vulnerable, and to prioritize research and interventions (37). Surveillance can help identify sentinel events and serve as an early warning of significant climate-related burdens (10). Existing national and corporate workplace surveillance systems should be assessed for their utility to indicate climate-related and work-specific information. However, new surveillance systems may be needed to capture climate-related sentinel events. Ultimately, surveillance data needs to be analyzed and regularly communicated to workers, employers, authorities, and the general public.

### Risk assessment

Risk assessment is critical to developing risk-management policies. Consequently there is a need for national policy-relevant and organizational assessments of occupational climate-related hazards. In particular, the potential for interactive nonlinear (when the output of a system does not change proportionally to its input) effects due to climate stressors needs to be assessed. Since climate-related risks arise due to compounding and cascading hazards and impacts, a cumulative risk assessment of climate, work, and lifelong risks may be necessary for worker protection (38). Ultimately, there is a need for new models that integrate work, health and safety (WHS) with climate data while also considering the impact of uncertainties, vulnerabilities, and resilience on risk assessments (10). Moreover, attribution science needs to be further developed, particularly as it applies to workers and the costs of the impact of climate events on them (39).

### Risk management

The objective of all these types of action (research, surveillance, and risk assessment) is to anticipate and manage risks by implementing mitigation and adaptation plans (40). Mitigation refers not only to reducing greenhouse gases but also to reducing the impact of exposure to climate-related hazards (41). Central to this objective is developing guidelines, policies, and standards to protect workers under changing climate conditions. As discussed in subsequent sections, it will be necessary to integrate WHS efforts with public health and authoritative efforts, particularly with regard to mitigation, adaptation, resilience, and preparedness. *The primary means to protect workers is by mitigating greenhouse gas (GHG) emissions.* However, globally, this mitigation is happening too slowly and not to a large enough extent (42, 43). These emissions are related to accelerated global warming, increased ambient temperature, air pollution, UV radiation, extreme weather, expanded habitats for disease vectors, and problems resulting from industrial transitions and the built environment (43). To adapt to ambient temperature increases, workers will need to avoid working during extreme heat hours. Policies, regulations, and new work practices must be developed to foster mitigation, adaptation, and system solutions to protect workers from these various climate-related hazards (16, 44, 45). Undergirding this action is the need for “a worker transition program that could assist workers adversely affected by climate change effects and policies” (45, 83).

## Action-modifying factors

The aforementioned preventive and control actions require supported decision-making by employers, authorities, workers, occupational health practitioners, politicians, and the general public. However, significant factors inhibit such action, including the lack of public awareness, acceptance, and support related to the threat and impact of climate change in general and on workers in particular (43). These public factors have mainly arisen due to the absence of political leadership and action and to the perpetration of deleterious political action (46, 47). The foundation for these factors that inhibit preventive action is built on climate misinformation, disinformation, denial, skepticism, distrust, lack of awareness, and delay (12, 48, 49). Indeed, the lack of public awareness and support for investment and action to mitigate climate change and aid both workers and the general public is a critical issue (43). There is a scientific consensus based on a century of scientific evidence that the Earth’s surface and its ocean basins are warming primarily due to the burning of fossil fuels (43, 50, 51). However, for example, more than a quarter of the US adult population believes global warming is due to natural patterns (52). In this belief environment, consistent leadership by officials and collective political action are not likely. It is not clear whether the general population, leaders, politicians, employers, and workers globally, understand the seriousness of the climate-change crisis, as illustrated in Table 1. Moreover, there is a vast absence of global political leadership that characterizes the climate-change situation as a universal problem requiring universal sacrifice. Politicians, scientists, and authorities need to be able to promote action for longer time horizons than the next round of elections as well as action that addresses the trade-off between individual versus collective benefits from controlling GHGs (42). This implies that every GHG emission by one stakeholder reduces the opportunity for a GHG emission by another (53). The recognition that GHG emissions are in fact competitive relative to the global carbon budget must be part of the solution (54). Addressing GHGs also requires accelerated transitions and sustainable replacements, such as renewable sources of energy. However, the real obstacles to such action are macroeconomic costs and distributional consequences leading to unequal impacts of climate-change mitigation (55, 56). Moreover, in addition to overcoming socioeconomic barriers, enabling factors need to be strengthened “to ensure the political economy can shift from being a brake on transformative climate actions to driving it.” (75; p 92).

**Table 1:** Selected effects indicative of the climate-change crisis.

Hazard	Effect
Ambient temperature	2024 was the warmest year in a 175-year observation period
Ambient temperature	Frozen parts of the earth are melting at an alarming rate
Carbon dioxide	Atmospheric concentrations are higher than any time in the last 2 million years
Methane, Nitrogen dioxide	Levels are higher than any time in past 800,000 years
Global sea level	Reached record-high levels
Ocean chemical composition	Getting more acidic

Adopted from the World Meteorological Association 2024 (82).

All of these observations and realizations have led to a delay of collective action to mitigate exposures and burdens among workers and the public. Generally, there appears to be a failure to greatly appreciate the impact that climate change has on workers, the public, and the environment (43, 47, 57, 58). Accordingly, evidence-based strategies, political pressure, and public health action to combat scientific misinformation and delayed action are urgently needed (59).

## New emphasis for worker protection against climate-related hazards

**Figure 3:**
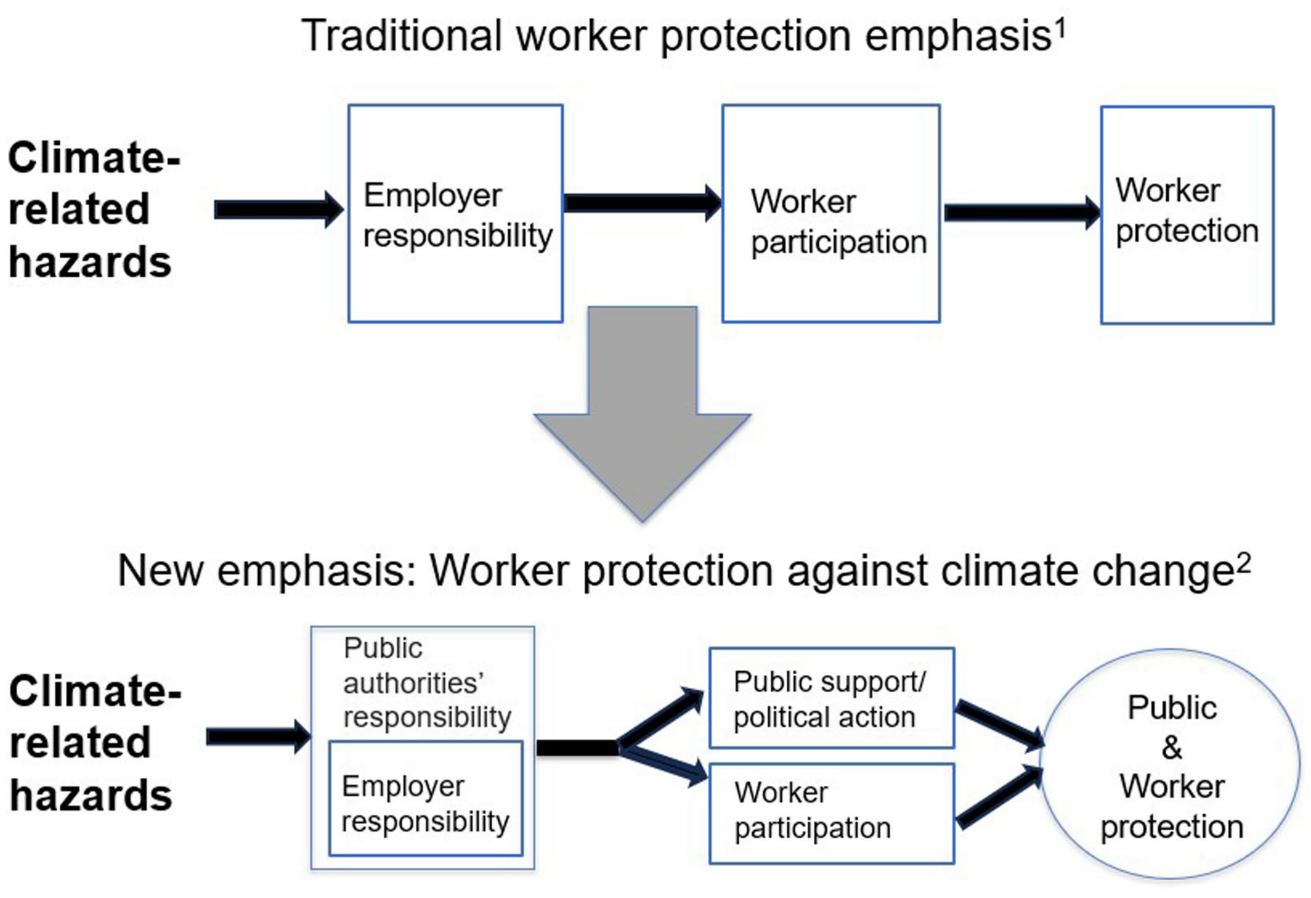
Transition to a new emphasis of worker protection against climate-related hazards. 1. Historically, employers have been responsible for protecting workers by controlling hazards. Workers have a responsibility to follow employers’ risk management guidance. 2. In the new emphasis, both employers and authorities have responsibility regarding climate-related hazards to workers. In addition to workers’ responsibility to follow employers’ risk management guidance there is a need for public support and political action to mitigate climate-related hazards to workers as well as the general public.

The lack of social and collective action to effectively address climate-related hazards drives the need for a new emphasis for worker protection against climate change (Figure 3). The emphasis illustrates that the responsibility for protecting workers from the adverse effects of climate change needs to transition from solely employers to both employers and public/political supporters. This dual responsibility is needed because climate change is not only a workplace issue but also a public issue that requires heightened political action to mitigate exposure, particularly in terms of fossil fuel use and GHG emissions (53). Achieving public support, political action, and worker support and participation will require addressing climate misinformation, disinformation, denial, and delay of mitigation. While these factors and lack of awareness are important elements that impede climate action, most of the world believes climate change is a real threat. The critical barrier appears to be arguments or discourses for delay of action. Lamb et al. (12) identified 12 typologies for this delay based on four questions: (1) is it our responsibility to take action? (2) Are transformative changes necessary? (3) Is it desirable to mitigate climate change given the costs? (4) Is it still possible to mitigate climate change? The answer to each of these questions is “yes.” There is a dual responsibility of mitigating climate change and protecting workers (60). However, to effectively approach each of these questions, the misinformation surrounding them needs to be resolved.

A growing literature argues that preemptively warning the public about misinformation can build resistance and “inoculate” against climate-change denial (12, 59, 61). However, there are also conflicting opinions about the value of such inoculation (62). Nevertheless, misinformation can and should be addressed through various preventive and follow-up techniques (63). Further, it should be understood that the new emphasis does not relieve employers from the responsibility of protecting workers from climate change. Rather, that responsibility is expanded because in addition to promoting adaptation and resilience protections, employers (i.e., businesses and corporations) need to become a stronger voice calling on politicians to act on mitigation policies, including addressing barriers to a scalable climate response (64, 65).

## Blueprint for the WHS field

**Figure 4:**
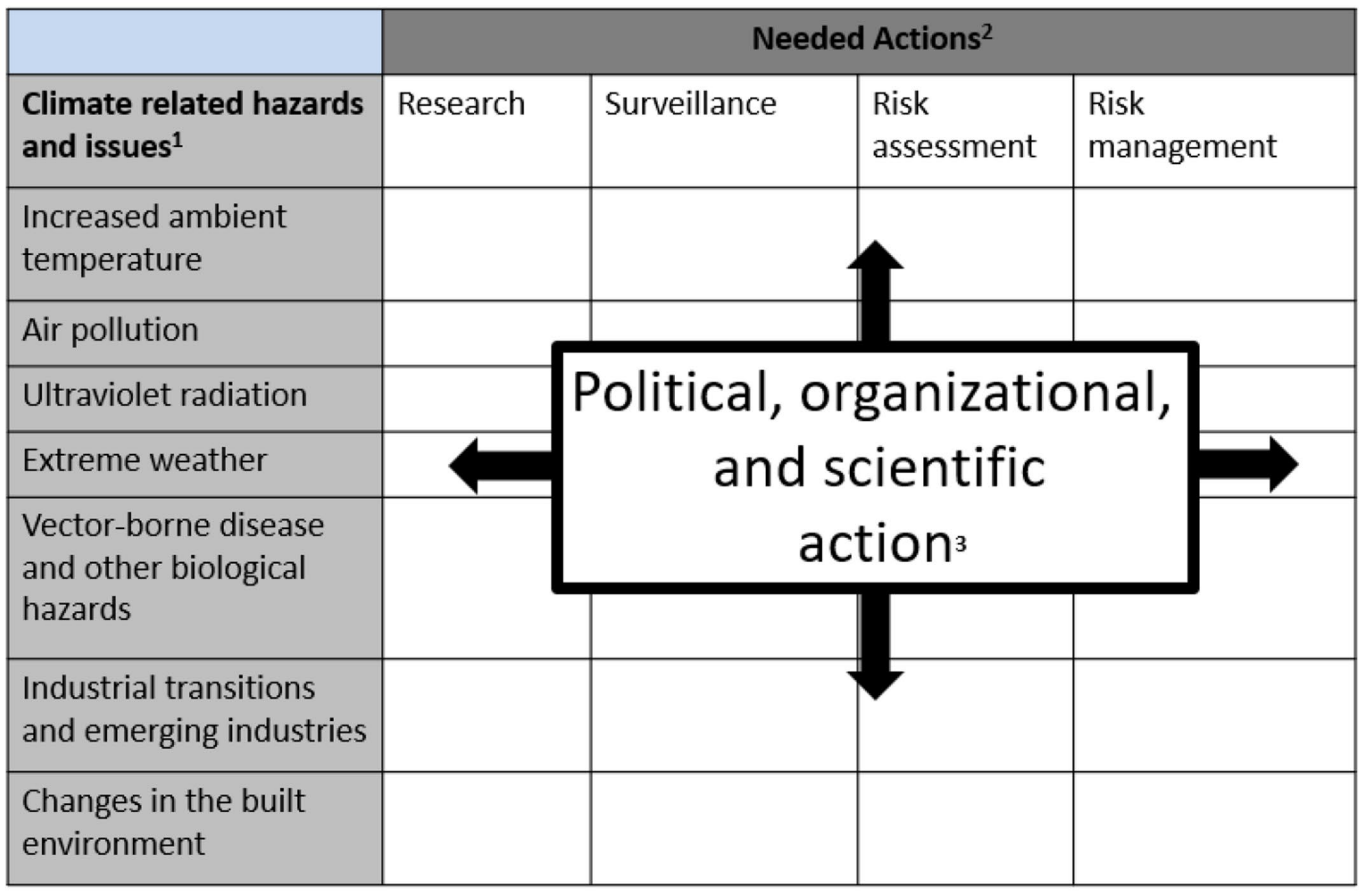
Blueprint for further action. 1. Climate-related hazards derived from references (6, 9, 10). 2. Needed actions described in categories that reflect both organizational and societal approaches to address WHS hazards (10, 33, 67, 68, 81). 3. The blank cells in the matrix provide focal points for political, organizational and scientific action to address climate-related hazards. Within each cell investigators, authorities, employers, and other stakeholders can set priorities, develop action plans and monitor progress.

If the framework (Figure 2) addressed in this paper is to be of use to protect workers, the WHS field will need to actively collaborate with communication professionals and social, political, implementation, and climate scientists to assess and address climate denial, skepticism, lack of awareness, complacency, misinformation, and delay (12, 30, 66–68). This collaboration can be accomplished by pursuing integrative action in each of the cells in the blueprint matrix in Figure 4. Additionally, the blueprint helps organize possible action that can be taken in each of the cells to investigate the etiology, magnitude, and impact of climate-related hazards; the effectiveness and sustainability of interventions for climate hazards; the development and implementation of control policies; and research on and interventions for action-inhibiting factors, such as misinformation, distrust, denial, and delay. For example, (looking at the first row of cells) if there is concern about increasing ambient temperature, investigators might conduct research on workers’ vulnerabilities or on public skepticism about anthropogenic causes of global warming; governments might develop climate-related surveillance programs; employers might assess workers’ risks from heat exposure and develop hazard management programs. Over all cells there is need to overcome climate misinformation by continually updating and adapting refutation strategies (67). Additionally, it is possible to utilize technological techniques that detect climate misinformation and counter it with generative based corrections (68). The blueprint is a means of prioritizing, promoting and monitoring action on climate-related hazards to workers.

## Urgency is the watchword

The efforts described in this Policy Brief must be undertaken expeditiously. The condition of the Earth is already dangerous in many places and nearing the tipping point (43, 69, 70). Numerous workers throughout the world are already suffering and dying from climate-related hazards (7, 8, 71). The primary need is to shift the public’s, workers’, decision-makers’, politicians’, and authorities’ opinions so that policies are enacted to mitigate GHG emissions and prevent exposure to the resultant climate-related hazards. Such action will protect not only the general population but workers as well. It is time to act to protect lives and the health of workers now and in the future.

## References

[1] Kiefer M Rodriguez-Guzman J Watson J Mergler D da Silva AS. Worker health and safety and climate change in the Americas: issues and research needs. Rev Panam Salud Publica. (2016) 40:192–7. Available at: https://iris.paho.org/handle/10665.2/31236 27991978 PMC5176103

[2] Kjellstrom T. Impact of climate conditions on occupational health and related economic losses: a new feature of global and urban health in the context of climate change. Asia Pac J Public Health. (2016) 28:28–37. doi: 10.1177/1010539514568711 25626424

[3] Levy BS Roelofs C. Impacts of climate change on workers’ health and safety. Oxford: Oxford research encyclopedias (2019).

[4] Constible J Chang B Morganelli C Blandon N. On the front lines: Climate change threatens the health of America’s workers. National Resources Defense Council. (2020). Available online at: https://www.nrdc.org/sites/default/files/front-lines-climate-change-threatens-workers-report.pdf (Accessed May 5, 2025)

[5] ANSES. Opinion of the French Agency for Food, environmental and occupational health and safety on the “assessment of the risks to worker health posed by climate change.” (2018). Available online at: https://www.anses.fr/en/system/files/AP2013SA0216EN.pdf (Accessed November 20, 2024)

[6] Schulte PA Jacklitsch BL Bhattacharya A Chun H Edwards N Elliott KC . Updated assessment of occupational safety and health hazards of climate change. J Occup Environ Hyg. (2023) 20:183–206. doi: 10.1080/15459624.2023.2205468, PMID: 37104117 PMC10443088

[7] Smith G. The world’s torrid future is etched in the crippled kidneys of Nepali workers. Washington post. (2023). Available online at: www.washingtonpost.com/world/2023/01/06/climate-change-heat-kidney-disease/ (Accessed November 20, 2024)

[8] ILO. (2024). Ensuring safety and health at work in a changing climate. Available online at: https://www.ilo.org/sites/default/files/2024-07/ILO_SafeDay24_Report_r11.pdf (Accessed May 5, 2024)

[9] Schulte PA Chun H. Climate change and occupational safety and health: establishing a preliminary framework. J Occup Environ Hyg. (2009) 6:542–54. doi: 10.1080/15459620903066008, PMID: 19551548

[10] Schulte PA Bhattacharya A Butler CR Chun HK Jacklitsch B Jacobs T . Advancing the framework for considering the effects of climate change on worker safety and health. J Occup Environ Hyg. (2016) 13:847–65. doi: 10.1080/15459624.2016.1179388, PMID: 27115294 PMC5017900

[11] US EPA. Priority climate action plans for states, MSAs, tribes, and territories. US Environmental Protection Agency. (2025). Available online at: https://www.epa.gov/inflation-reduction-act/priority-climate-action-plans-states-msas-tribes-and-territories (Accessed May 15, 2025)

[12] Lamb WF Mattioli G Levi S Roberts JT Capstick S Creutzig F. Discourses of climate delay. Glob Sustain. (2020) 3:e17. doi: 10.1017/sus.2020.13

[13] MacInnis DJ. A framework for conceptual contributions in marketing. J Mark. (2011) 75:136–54. doi: 10.1509/jmkg.75.4.136

[14] Romanello M Di Napoli C Drummond P. The 2022 report of the lancet countdown on health and climate change: health at the mercy of fossil fuels. Lancet. (2022) 400:1619–54. doi: 10.1016/S0140-6736(22)01540-9, PMID: 36306815 PMC7616806

[15] Jaggernath J Haslam D Naidoo KS. Climate change: impact of increased ultraviolet radiation and water change on eye health. Health (San Francisco). (2013) 5:921. doi: 10.4236/health.2013.55122

[16] NIOSH. Criteria for a recommended standard: Occupational exposure to heat and hot environments. (2016). Available online at: https://www.cdc.gov/niosh/docs/2016-106/default.html (Accessed November 20, 2024)

[17] Spector JT Masuda YJ Wolff NH Calkins M Sexias N. Heat exposure and occupational injuries. Review of the literature and implications. Curr environ. Health Rep. (2019) 6:286–96. doi: 10.1007/s40572-019-00250-8, PMID: 31520291 PMC6923532

[18] Romps DM Seely JT Vollaro D Molinari J. Projected increase in lightning strikes in the United States due to global warming. Science. (2014) 346:851–4. doi: 10.1126/science.1259100, PMID: 25395536

[19] Vega-Calderón L Almendra R Fdez-Arroyabe P Zarrabeitia MT Santurtún A. Air pollution and occupational accidents in the community of Madrid, Spain. Int J Biometeorol. (2020) 65:429–36. doi: 10.1007/s00484-020-02027-3, PMID: 33029653

[20] Fayard GM. Fatal work injuries involving natural disasters, 1992-2006. Disaster Med Public Health Prep. (2009) 3:201–9. doi: 10.1097/DMP.0b013e3181b65895, PMID: 20081416

[21] Engkvist I Swensson R Eklund J. Reported occupational injuries at Swedish recycling centres - based on official statistics. Ergonon. (2011) 54:357–66. doi: 10.1080/00140139.2011.556261, PMID: 21491278

[22] Brooks SK Greenberg N. Climate change effects on mental health: are there workplace implications. Occup Med. (2023) 73:133–7. doi: 10.1093/occmed/kqac100, PMID: 36170162 PMC10132205

[23] Crandon TJ Dey C Scott JG Thomas HJ Ali S Charlson FJ. The clinical implications of climate change for mental health. Nat Hum Behav. (2022) 6:1474–81. doi: 10.1038/s41562-022-01477-6, PMID: 36385181

[24] Obradovich N Miglorini R Paulus MP Rahwan L. Empirical evidence of mental health risks posed by climate change. PNAS. (2018) 115:10953–8. doi: 10.1073/pnas.180152811 30297424 PMC6205461

[25] Varghese B Hansen A Bi P Pisaniello D. Are workers at risk of occupational injuries due to heat exposure? A comprehensive literature review. Saf Sci. (2018) 110:380–92. doi: 10.1016/j.ssci.2018.04.027

[26] Mao X Fung OWM Hu X Loke AY. Psychological impacts of disaster on rescue workers: a review of the literature. Int J Disaster Risk Reduct. (2018) 27:602–17. doi: 10.1016/j.ijdrr.2017.10.020

[27] Nolan MS Hause AM Murray KO. Findings of long-term depression up to 8 years post infection from West Nile virus. J Clin Psychol. (2012) 68:801–8. doi: 10.1002/jclp.21871, PMID: 23929558 PMC6211791

[28] Conroy G. ‘Ecological grief’ grips scientists. Underw Nat. (2019) 573:318–9. doi: 10.1038/d41586-019-02656-8, PMID: 31530920

[29] Guo J Kubli D Saner P. The economics of climate change: no action not an option. Swiss Re Institute (2021). Available online at: https://www.swissre.com/dam/jcr:e73ee7c3-7f83-4c17-a2b8-8ef23a8d3312/swiss-re-institute-expertise-publication-economics-of-climate-change.pdf (Accessed November 20, 2024)

[30] Eurofound. Impact of climate change and climate policies on living conditions, working conditions, employment and social dialogue: A conceptual framework Publication Office of the European Union Luxembourg (2023).

[31] Kotz M Levermann A Wenz L. The economic commitment of climate change. Nature. (2024) 628:551–7. doi: 10.1038/s41586-024-07219-0, PMID: 38632481 PMC11023931

[32] WHO. The social dimensions of climate change: Discussion draft. Geneva: World Health Organization (2021).

[33] International Labor Organization. Plan safe, plan healthy: Guidelines for developing National Programmes on occupational safety and health. Geneva: ILO (2013).

[34] Agache I Hernandez ML Radbel JM Renz H Akdis CA. An overview of climate changes and its effects on health: from mechanism to one health. J Allergy Immunol: Practice. (2025) 13:253–64. doi: 10.1016/j.jaip.2024.12.025 39725316

[35] World Health Organization. Climate change. (2023). Available online at: https://www.who.int/news-room/fact-sheets/detail/climate-change-and-health (Accessed October 15, 2025)

[36] Neta G Pan W Ebi K. Advancing climate change health adaptations through implementation science. Lancet Planet Health. (2022) 6:e909–18. doi: 10.1016/S2542-5196(22)00199-1 36370729 PMC9669460

[37] Palmeiro-Silva Y Aravena-Contreras R Gana JI Tapia RS Kelman I. Climate-related health impact indicators for public health surveillance in a changing climate: a systematic review and local sustainability analysis. Lancet Reg Health Am. (2014) 38:100854. doi: 10.1016/j.lana.2024.100854 PMC11334688 39171197

[38] UNDRR. Technical guidance on comprehensive risk assessment and planning in the context of climate change. United Nations Office for disaster risk reduction. (2022). Available online at: https://www.undrr.org/publication/technical-guidance-comprehensive-risk-assessment-and-planning-context-climate-change (Accessed May 5, 2025)

[39] National Academies of Science, Engineering and Medicine. Attribution of extreme weather events in the context of climate change. Washington DC: The National Academies Press (2016).

[40] Ansah EW Amoadu M Obeng P Sarfo O. Health systems response to climate change adaptation: a scoping review of global evidence. BMC Public Health. (2024) 24:215. doi: 10.1186/s12889-024-19459-w, PMID: 39075368 PMC11285469

[41] Stults M. Integrating climate change into hazard mitigation planning. Clim Risk Manag. (2017) 17:21–34. doi: 10.1016/j.crm.2017.06.004

[42] Montfort S Fresenfeld L Ingold K Lamb WF Andrijevic M. Political enablers of ambitious climate policies: a framework and thematic review. NPJ Clim Action. (2025) 4:14. doi: 10.1038/s44168-024-00206-1 39959135 PMC11828736

[43] Hansen JE Kharecha P Sato M Tselioudis G Kelly J Bauer SE . Global warming has accelerated: are the United Nations and the public well-informed? Environ Sci Policy Sustain Dev. (2025) 67:6–44. doi: 10.1080/00139157.2025.2434494

[44] ISO. ISO 14090:2019. Adaptation to climate change—principles, requirements and guidelines. International Organization for Standardization. (2019). Available online at: https://www.iso.org/standard/68507.html (Accessed November 20, 2024)

[45] Open Knowledge Repository. Reality check: Lessons from 25 policies advancing a low-carbon future. World Bank Group. (2023). Available online at: https://openknowledge.worldbank.org/handle/10986/40262 (Accessed November 20, 2024)

[46] Dupuis J. Political barriers to climate change adaptation. United Nations University, our world. (2011). Available online at: https://ourworld.unu.edu/en/political-barriers-to-climate-change-adaptation (Accessed November 20, 2024)

[47] Kamarck E. The challenging politics of climate change Brookings Institution (2019).

[48] Gounaridis D Newell JP. The social anatomy of climate change denial in the United States. Sci Rep. (2024) 14:2097. doi: 10.1038/s41598-023-50591-6, PMID: 38355774 PMC10866916

[49] Hornsey MJ Lewandowsky S. A toolkit for understanding and addressing climate skepticism. Nat Hum Behav. (2022) 6:1454–64. doi: 10.1038/s41562-022-01463-y 36385174 PMC7615336

[50] NASA. Scientific consensus. (2024). Available online at: https://science.nasa.gov/climate-change/scientific-consensus/ (Accessed November 20, 2024)

[51] IPCC. Summary for policymakers In: Masson-Delmotte et al. (eds). Climate change. The physical science basis. Contribution of working group 1 to the sixth assessment report of the intergovernmental panel on climate change. Cambridge, UK: Cambridge University Press (2021). 3–32.

[52] Pasquini G Spencer A Tyson A Funk C. Why some Americans do not see the urgency on climate change Pew Research Center (2023).

[53] Meckling J Karplus VJ. Political strategies for climate and environmental solutions. Nat Sustain. (2023) 6:742–51. doi: 10.1038/s41893-023-01109-5

[54] Lane M. Political theory on climate change. Annu Rev Polit Sci. (2016) 19:107–23.

[55] Hodok J Kozluh T. Distribution impacts of energy transition pathways and climate change. OECD economics department working papers no. 1820 OECD Publishing (2024).

[56] Dervis K Strauss S. The real obstacle to climate action. Project syndicate. (2019). Available online at: https://www.brookings.edu/articles/the-real-obstacle-to-climate-action (Accessed September 22, 2025)

[57] Gallup. Are Americans concerned about global warming? The short answer. (2024). Available at: https://news.gallup.com/poll/355427/americans-concerned-global-warming.aspx

[58] Blackmon D. NIMBYism is global, and that’s a problem for energy transition. Forbes. (2022). Available online at: https://www.forbes.com/sites/davidblackmon/2022/01/23/nimbyism-is-global-and-thats-a-problem-for-the-energy-transition/ (Accessed May 5, 2025)

[59] Farrell J McConnell K Brulle K. Evidence-based strategies to combat scientific misinformation. Nat Clim Chang. (2019) 9:191–5.

[60] Iskander NN Lowe N. Climate change and work: politics and power. Annu Rev Polit Sci. (2020) 23:111–31. doi: 10.1146/annurev-polisci-061418-095236

[61] Roozenbeek J van der Linden S Goldberg B Rathje S Lewandowsky S. Psychological inoculation improves resilience against misinformation on social media. Sci Adv. (2022) 8:eabo6254. doi: 10.1126/sciadv.abo6254, PMID: 36001675 PMC9401631

[62] Kupferschmidt K. You won’t believe this. Science. (2024) 386:483–5. doi: 10.1126/science.adu2101, PMID: 39480939

[63] Lewandowsky S Cook J Ecker U The debunking handbook 2020. (2020).

[64] Hallegatte S Godinho C Rentschler J. Within reach: Navigating the political economy of decarbonization. Climate change and development series Washington DC: World Bank (2023).

[65] Tomer A Kane J Singer A Mobilizing the market: The barriers to financing a more scalable climate response. Brookings metro. (2024). Available online at: https://www.brookings.edu/articles/mobilizing-the-market-the-barriers-to-financing-a-more-scalable-climate-response/ (Accessed October 10, 2025).

[66] Heneghan J John DC Bartsch SM. A system map of the challenges of climate communication. J Health Commun. (2024) 29:77–88. doi: 10.1080/10810730.2024.2361842 38845202 PMC11414781

[67] Ulrich A. Climate misinformation: communicating climate science in an era of misinformation. Intersect. (2022) 16:1–28. Available at: https://ojs.stanford.edu/ojs/index.php/intersect/article/view/2395

[68] Zanartu F Cook J Wagner M Garus J. A technocognitive approach to detecting fallacies in climate misinformation. Nat Sci Rep. (2024) 14:27647. doi: 10.1038/s41598-024-76139-w PMC11557567 39532907

[69] Wunderling N von der Heydt AS Aksenov Y Barker S Bastiaansen R Brovkin V . Climate tipping point interactions and cascades: a review. Earth Syst Dynam. (2024) 15:41–74. doi: 10.5194/esd-15-41-2024

[70] UNDP. What is climate change mitigation and why is it urgent? United Nations development Programme. (2024). Available online at: https://climatepromise.undp.org/news-and-stories/what-climate-change-mitigation-and-why-it-urgent (Accessed November 20, 2024)

[71] Wuersch L Neher A Marino FE Bamberry L Pope R. Impacts of climate change on work health and safety in Australia: a scoping literature review. Int J Environ Res Public Health. (2023) 20:7004. doi: 10.3390/ijerph20217004, PMID: 37947561 PMC10650313

[72] National Assessment Synthesis Team. Overview In: Climate change impacts on the United States: The potential consequences of climate variability and change. US Global Change Research Program, Washington DC, 2000: Cambridge University Press (2001). 620.

[73] Karl TR Melillo JM Petersen TC. Global climate change impacts in the United States. Washington DC: US Global Change Research Program (2009).

[74] Melillo JM Richmonds TC Yohe GW. Climate change impacts in the unites states: The third national assessment. Washington DC: US Global Change Research Program (2014).

[75] Reidmiller DR Avery CW Easterling DR. Fourth national climate assessment. Volume ii: Impacts, risks, and adaptation in the United States US Global Research Program, Washington DC (2017).

[76] Crimmins AR Avery CW Easterling DK. Fifth national climate assessment US Global Research Program, Washington DC (2023).

[77] McCarthy JJ Leary NH Dokken DJ White KS. Climate change 2001: Impacts, adaptation, and vulnerability. Contribution of working group ii to the third assessment report of the intergovernmental panel on climate change. Cambridge, UK: Cambridge University Press (2001).

[78] Parry ML Palutikof JP van der Linden PJ Hanson CE. Climate change 2007: Impacts, adaptation and vulnerability. Contribution of working group ii to the fourth assessment report of the international panel on climate change Cambridge University Press (2007).

[79] Field CB Barros VR Dokken DJ. Climate change 2014: Impacts, adaptation, and vulnerability. Part a. global and sectorial aspects. Contribution of working group ii to the fifth assessment report of the international panel on climate change Cambridge University Press (2014).

[80] Pörtner HO Roberts DC Tiqnor M. Climate change 2022: Impacts, adaptation, and vulnerability. Global and sectoral aspects. Contribution of working group ii to the fifth assessment report of the international panel on climate change Cambridge University Press (2022).

[81] Safe Work Australia. (n.d.). Managing risks. Available online at: https://www.safeworkaustralia.gov.au/safety-topic/managing-health-and-safety/identify-assess-and-control-hazards/managing-risks.

[82] World Meteorological Organization. State of the global climate 2024. (2025). Available online at: https://wmo.int/publication-series/state-of-global-climate-2024 (Accessed May 5, 2025)

[83] Barrett J. Worker transition and global climate change. Report prepared for the PEW center on global climate change. (2001). Available online at: https://www.c2es.org/document/worker-transition-global-climate-change/ (Accessed May 5, 2025)

