# Correction: Framework for developing research and policy to address climate-related occupational hazards

**DOI:** 10.3389/fpubh.2026.1812372

**Published:** 2026-03-09

**Authors:** Paul A. Schulte

**Affiliations:** Independent Occupational Safety and Health Consultant, Cincinnati, OH, United States

**Keywords:** climate change, ambient temperature, workers, environment, public health

In the **Acknowledgments**, the name of Frank Pot was inadvertently omitted from the list of those thanked. In addition, Brenda Jacklitsch's name was incorrectly spelled as Jacklitch. The correct sentence reads:

“The author thanks Frank Pot, Lee Newman, Gregory Wagner, and Brenda Jacklitsch for comments on earlier versions of the paper, and Antje Ruppert and Ali Ferguson for technical support.”

The original version of this article has been updated.

